# Supplementary material for: Bioconversion of Furanic Compounds by Chlorella vulgaris—Unveiling Biotechnological Potentials
Source: Microorganisms. 2024 Jun 18;12(6):1222. doi: 10.3390/microorganisms12061222 (PMC11205514; doi:10.3390/microorganisms12061222)
Supplement: Supplementary file 1 [file microorganisms-12-01222-s001.zip › microorganisms-3055949-supplementary.pdf]

Table S1: BLAST results from matching the known enzymes to the *Chlorella vulgaris* Genome

Reference	Organism	Enzyme	Gene	BLAST						
				Max Score	Total Score	Query Cover (%)	E value	Identity (%)	Accession Length	Accession
[14]	*Pseudomonas putida*	HMF/furfural oxidoreductase	hmfA	44,3	44,3	5	0,0006	41,18	5048413	SIDB01000002.1
			hmfB	no significant similarity found						
			hmfC	38,9	38,9	44	0,001	33,77	5048413	SIDB01000002.1
			hmfD	40,8	40,8	7	0,003	47,22	2713456	SDIB01000003.1
			hmfE	44,7	44,7	15	0,00005	48,78	1687577	SIDB01000013.1
			hmfF	no significant similarity found						
			hmfG	no significant similarity found						
[19]	*Cupriavidus basilensis*	HMF furfural oxidoreductase	hmfA	40,4	40,4	5	0,008	37,93	5048413	SIDB01000002.1
			hmfB	no significant similarity found						
			hmfC	38,1	38,1	54	0,003	37,61	5048413	SIDB01000002.1
			hmfD	42,7	42,7	6	0,0007	55,56	2713456	SDIB01000003.1
			hmfE	44,7	44,7	14	0,00005	52,63	1687577	SIDB01000013.1
			hmfF	no significant similarity found						
			hmfG	no significant similarity found						
			hmfH	87,8	183	41	1E-17	39,42	5048413	SIDB01000002.1
